# Atypical Ataxia Presentation in Variant Ataxia Telangiectasia: Iranian Case-Series and Review of the Literature

**DOI:** 10.3389/fimmu.2021.779502

**Published:** 2022-01-14

**Authors:** Tannaz Moeini Shad, Reza Yazdani, Parisa Amirifar, Samaneh Delavari, Marzieh Heidarzadeh Arani, Seyed Alireza Mahdaviani, Mahnaz Sadeghi-Shabestari, Asghar Aghamohammadi, Nima Rezaei, Hassan Abolhassani

**Affiliations:** ^1^ Research Center for Immunodeficiencies, Pediatrics Center of Excellence, Children’s Medical Center, Tehran University of Medical Sciences, Tehran, Iran; ^2^ Primary Immunodeficiency Diseases Network (PIDNet), Universal Scientific Education and Research Network (USERN), Tehran, Iran; ^3^ Department of Neurology, Thomas Jefferson University, Philadelphia, PA, United States; ^4^ Department of Medical Genetics, School of Medicine, Tehran University of Medical Sciences, Tehran, Iran; ^5^ Department of Asthma, Allergy & Immunology, Kashan University of Medical Sciences, Kashan, Iran; ^6^ Pediatric Respiratory Diseases Research Center, National Research Institute of Tuberculosis and Lung Diseases (NRITLD), Shahid Beheshti University of Medical Sciences, Tehran, Iran; ^7^ Immunology Research Center, Tabriz University of Medical Sciences, Tabriz, Iran; ^8^ Network of Immunity in Infection, Malignancy and Autoimmunity (NIIMA), Universal Scientific Education and Research Network (USERN), Tehran, Iran; ^9^ Division of Clinical Immunology, Department of Biosciences and Nutrition, Karolinska Institutet, Huddinge, Sweden; ^10^ Division of Clinical Immunology, Department of Laboratory Medicine, Karolinska Institute at Karolinska University Hospital Huddinge, Stockholm, Sweden

**Keywords:** primary immunodeficiency, inborn errors of immunity, ataxia-telangiectasia, late-ataxia, atypical presentation, mild phenotype, autoimmunity

## Abstract

Ataxia-telangiectasia (AT) is a rare autosomal recessive neurodegenerative multisystem disorder. A minority of AT patients can present late-onset atypical presentations due to unknown mechanisms. The demographic, clinical, immunological and genetic data were collected by direct interview and examining the Iranian AT patients with late-onset manifestations. We also conducted a systematic literature review for reported atypical AT patients. We identified three Iranian AT patients (3/249, 1.2% of total registry) with later age at ataxia onset and slower neurologic progression despite elevated alpha-fetoprotein levels, history of respiratory infections, and immunological features of the syndrome. Of note, all patients developed autoimmunity in which a decrease of naïve T cells and regulatory T cells were observed. The literature searches also summarized data from 73 variant AT patients with atypical presentation indicating biallelic mild mutations mainly lead to an atypical phenotype with an increased risk of cancer. Variant AT patients present with milder phenotype or atypical form of classical symptoms causing under- or mis- diagnosis. Although missense mutations are more frequent, an atypical presentation can be associated with deleterious mutations due to unknown modifying factors.

## Introduction

Ataxia-telangiectasia (AT) is a rare autosomal recessive multisystem disorder caused by mutations in the *ataxia telangiectasia mutated (ATM)* gene encoding a protein kinase with a key role in the cell cycle control and DNA repair (1, 2). Given the residual kinase activity and the type of *ATM* mutation, the clinical spectrum of AT varies from a severe (early-onset and rapidly progressing neurodegeneration classic) phenotype to a variant atypical form (a relatively mild neurological phenotype and a lower risk of systemic complications) (3).

Classical AT is characterized by early childhood-onset cerebellar ataxia, telangiectasia, immunodeficiency, increased serum alpha-fetoprotein (AFP) levels, autoimmune or other chronic inflammatory diseases, radiosensitivity, and increased susceptibility to malignancies. Most children are usually wheelchair-bound by age 10 years and die in the second decade of life due to malignancies or respiratory failure (4–6). In contrast to the classical AT group, variant AT patients with milder late-onset atypical phenotypes characterized by a predominance of extrapyramidal features (instead of cerebellar), late ataxia onset, slower neurologic progression with a mild phenotype, and an extended lifespan. Respiratory diseases and immunodeficiency are less severe in variant AT; however, the risk of malignancy is likely high, and such patients may also be radiosensitive (7–9). Overall, the classical form is caused by biallelic deleterious *ATM* mutations, which lead to a total loss of ATM protein, while majority of patients with milder phenotypes carry at least one missense or leaky splice site mutation (still producing the protein with residual function or kinase activity) (8). Moreover, some reports suggest that besides residual ATM kinase activity, other factors such as modifying genes and environmental factors might be involved in the presentation of milder phenotypes of AT (10).

Here, we describe three Iranian AT patients with variant atypical phenotypes who presented late-onset manifestations and developed autoimmunity in the course of the disease. For the first time, we reviewed systematically the literature of all previously reported AT atypical patients who had non-ataxia dominant signs or had late-onset ataxia presentation to illustrate a comprehensive picture of the milder AT phenotype.

## Materials and Methods

### Patients and Clinical Evaluation

Data of new patients including demographic data, medical history, and physical examination, were collected by medical records through national consensus on diagnosis and management guidelines for primary immunodeficiency (PID) (11). Demographic data included age, gender, age at disease onset, and age of diagnosis. The laboratory data were complete cell blood counts, serum AFP level, T- and B-cells subsets, and serum levels of immunoglobulins as previously described (12, 13). Medical information was collected after obtaining written informed consent from the patients and their parents, following the principles of the ethics committee of the Tehran University of Medical Sciences (12, 14). Diagnosis of the patients was based on the European Society for Immunodeficiency (ESID) guideline (15), including ataxia and at least two of the following: oculocutaneous telangiectasia, elevated AFP, lymphocyte AT karyotype with translocation chromosome 7:14, and cerebellar hypoplasia on magnetic resonance imaging (MRI). The whole-exome sequencing and confirmatory Sanger sequencing method were carried out according to a pipeline published previously (16–20), and a radiosensitivity test using G0 and G2 phase assays on phytohemagglutinin (PHA) stimulated peripheral blood lymphocytes were performed (21).

### Search Strategy for Reported Variant AT Patients

The literature searches for all reported atypical AT patients who had no dominant ataxia signs or had late-onset (≥ 5 years) ataxia reported up to September 1, 2021, was conducted by PubMed, Web of Science, and Scopus databases, applying the following keywords: “ataxia-telangiectasia” OR its synonyms (“ATM” OR “Louis-Bar syndrome”) in combination with terms for unusual ataxia presentation (including “late-onset” OR “variant” OR “adolescence” OR “atypical” OR “mild”). The articles were first screened based on the title and abstract to exclude all irrelevant studies, and then their full-text manuscripts were assessed for eligibility criteria (written in English, conducted on human subjects, reporting at least one patient with unusual ataxia presentation and detailed description of genetic, clinical, and immunological features). A manual search of bibliographies of the retrieved publications was performed to identify additional relevant studies (**Supplementary Figure 1**). All cases were categorized into three neurological phenotypic groups, based on the presence of ataxia and using a wheelchair. Group I, Mild for patients without dominant ataxic signs. Group II, Moderate for patients who developed late ataxia but without being wheelchair-bound. Group III, Severe for patients who developed late ataxia and used a wheelchair. Central and descriptive statistics were reported for quantitative data. For variables with non-normal distribution, median and interquartile ranges (IQR) were reported. All statistical tests were 2 tailed, and a *P*-value of less than 0.05 was considered statistically significant. All statistical analyses were performed using the SPSS software (v. 26.0, Chicago, IL).

## Results

### New Case Presentations

The first atypical AT patient in the Iranian PID registry is a 9-year-old boy, the only child of a consanguineous parent. The patient had a healthy situation until nine months and then he was referred to the Children’s Medical Center, Tehran, Iran, due to recurrent respiratory infections. In the maternal branch of the pedigree, typical AT was evident in his cousin. The patient manifested several episodes of oral aphthous during the first year of age, but it was not further evaluated. At the age of 1 year, he initiated walking without ataxia. Five months later, he was hospitalized due to a 10-day fever and neutropenia and suspected to Kawasaki disease, and treatment with intravenous immunoglobulin (IVIG) 400 mg/kg for 3 weeks was started. Over the first 2 years of his life, he was also admitted 3 times due to fever and acute sinusitis. At the age of 3 years, he presented petechiae and bleeding in the oral cavity due to idiopathic thrombocytopenic purpura (ITP), well responded to IVIG and corticosteroids. The patient was hospitalized for one week because of chickenpox and neutropenia at the age of 4 years. A diagnosis of AT was fulfilled by presenting clinical manifestations (ataxia, nystagmus, and ocular telangiectasia), laboratory findings (increased serum AFP) and genetic testing (a homozygous c.3895delG, p.A1299Pfs*50; frameshift mutation of the *ATM* gene) at the age of 6 years. Other immunologic and laboratory data are provided in **Supplementary Table 1**. At 7 years of age, the patient was hospitalized for parotitis, and presented skin vesicular lesions and shingles. He was discharged after the successful treatment of herpes zoster and his neurologic status is stable without prophylactic treatment.

**Figure 1:**
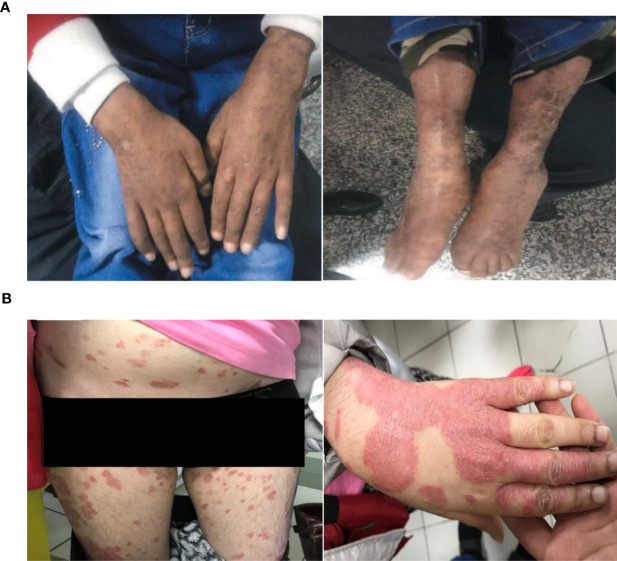
**(A)** Cutaneous granulomatous lesions on the extremities (Case 2); **(B)** extensive skin lesions of plaque psoriasis, which coalesce to cover almost the whole of the body surface (Case 3).

The second patient is a 13-year-old girl, with a history of parental consanguinity without immunodeficiency in her family, and she had normal developmental milestones. At the age of 3 years, she was admitted to the hospital due to thrombocytopenia, spontaneous bruising, and purpura. She was diagnosed with ITP, and then was treated with prednisolone for two years. At the age of 6 years, she was hospitalized due to worsening skin lesions. A thigh skin lesion and bone marrow biopsy revealed granulomatous inflammation (**Figure 1A**). At this age, she presented ataxic gait and telangiectasia as well as splenomegaly. Further neurological examination demonstrated strabismus and a slight head and trunk tremor, as well as dystonia and dysarthria. Increased AFP and presence of a homozygous large deletion variant spanning in exons 37–48 of the *ATM* gene confirmed diagnosis of AT at the age of 12 years. Immunologic and laboratory data are mentioned in detail in **Supplementary Table 1**. At this age, she had an eye infection, otitis media, and hearing loss. Unfortunately, the patient deceased at the age of 13 due to respiratory infections, despite mild neurological complications.

The third patient recorded is a 24-year-old female who was born full term as the third child of a consanguineous parent with no complications. Family history was remarkable for the second 16-year-old brother, who died because of a pulmonary infection as a result of AT. As the first clinical manifestation, the case presented a moderate ataxic gait and mild ocular telangiectasia at the age of 8 years, while other neurological examinations were normal. Due to disability in movement and dysarthria, she had to leave her education at 12 years of age. She was fully immunized with no complications by the age of 22 years. At this age, she presented with stable, severe, itchy, and quite sore plaques covered by scaly lesions. Several topical treatments were ineffective over the past year. Skin biopsy showed psoriasis disease (**Figure 1B**), and she started to receive adalimumab injection (loading by 80 mg then followed by 40 mg every other week since week 1). Laboratory tests showed diminished levels of IgA and an elevated AFP level (411 mg/dL mg/dL). She also showed increased radiosensitivity. Data of other immunological findings are summarized in **Supplementary Table 1**. The diagnosis of AT was confirmed by identifying a homozygous stop-gain mutation (c.6658C>T, p.Q2220*). Currently, the patient is ambulant, does not require walking aids, and is maintaining the same clinical and laboratory conditions.

Of note, none of these patients have other damaging mutations in correct inheritance patterns in other known PID genes classified based on the International Union of Immunological Societies (IUIS) expert committee (22, 23).

### Literature Review and Demographic Features of Atypical AT Patients

Our literature search revealed 73 variant AT patients with unusual ataxia phenotype (58% females) (**Supplementary Tables 2**, **3**). The median (IQR) age at onset was 11 (5.7-17) years, as males had a slightly earlier age onset of 10 (6 -22) years compared with females 12 (1.2-17) years. The first presentation of one-third of patients (29%) was after age 14 years, while 9 patients had the first presentation after 20 years of age. A minority of patients were classified in the mild group (n=16), however moderate (n=32) and severe (n=19) cases were more frequent. Classification of disease severity was not available for six cases due to a lack of data. Atypical patients in the severe group had significantly lower median (IQR) age of disease onset 6.5 (2.3-9.7) years compared to moderate 17 (6-29.5, *p*=0.001) years, and mild 11 (2.6-16.5, *p*=0.04) years, groups.

### Neurological features of Atypical AT Patients

**Figure 2:**
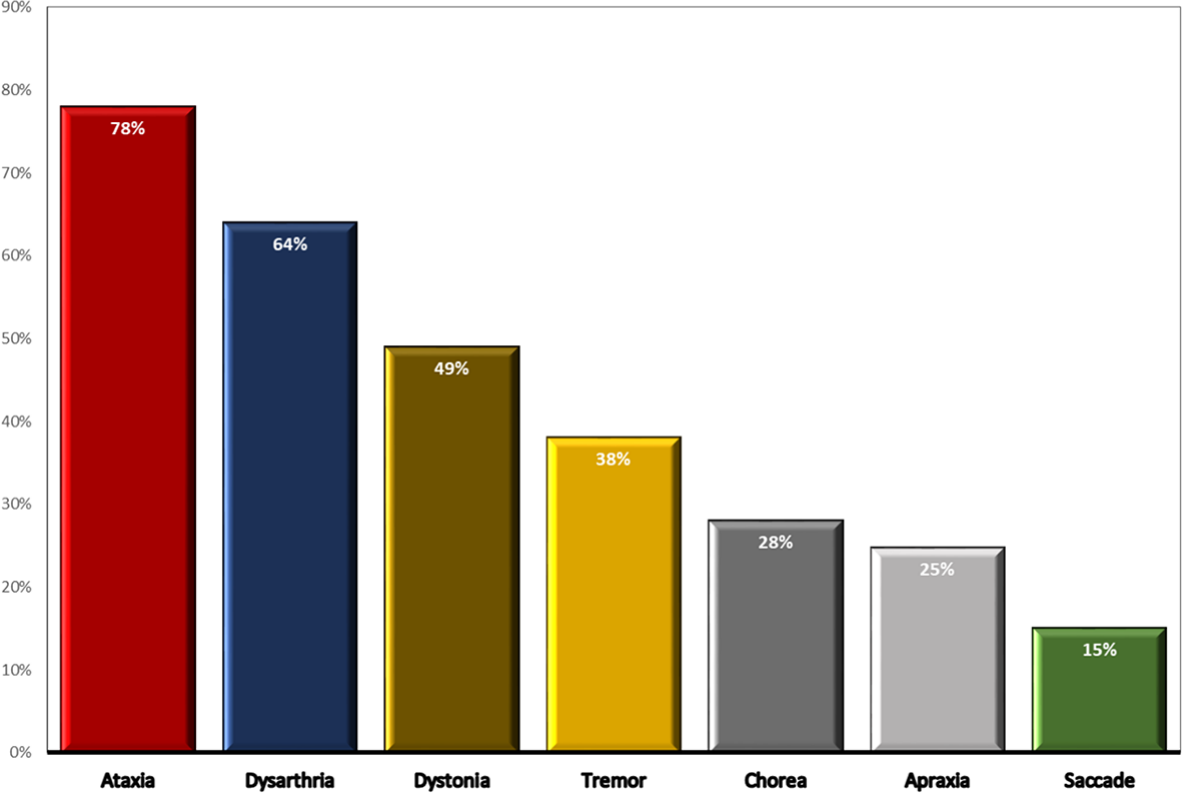
Comparison of neurological features of the 73 described variant AT patients.

Late presentation of cerebellar ataxia was presented in 78% (n=57) patients with a median (IQR) age at onset of 13 (7- 28) years, while 22% (n=16) of patients had no dominant ataxic signs. In the severe group, the median (IQR) age at onset of ataxia was significantly lower than the moderate group [8 (6.5-13) vs. 27 (17-33) years, *p*=0.001]. Among recorded neurological complications, dysarthria (64%), dystonia (49%), chorea (28%), and tremors (38%) were the most common. Oculomotor abnormalities in the form of saccades and oculomotor apraxia have been observed in 15% and 24.7%, respectively. Seven individuals got their first wheelchair within 10-20 years of symptom onset, while 6 cases required it after 20 years. Approximately 75% of patients presented extrapyramidal features including dystonia, tremors, and chorea movements. Cerebellar atrophy was found in 35 (65%) individuals at age of 28 (22-31) years, while 19 cases demonstrated normal imaging of the cerebellum. Expectedly, the frequency of cerebellar atrophy was higher in the severe group (83.8%) than the moderate (70.3%; *p*=0.6) and mild (38.4%; *p*=0.01) groups. Comparison of neurological features of the 73 described variant AT patients is illustrated in **Figure 2**.

### Clinical and Immunological Features of Atypical AT Patients

**Figure 3:**
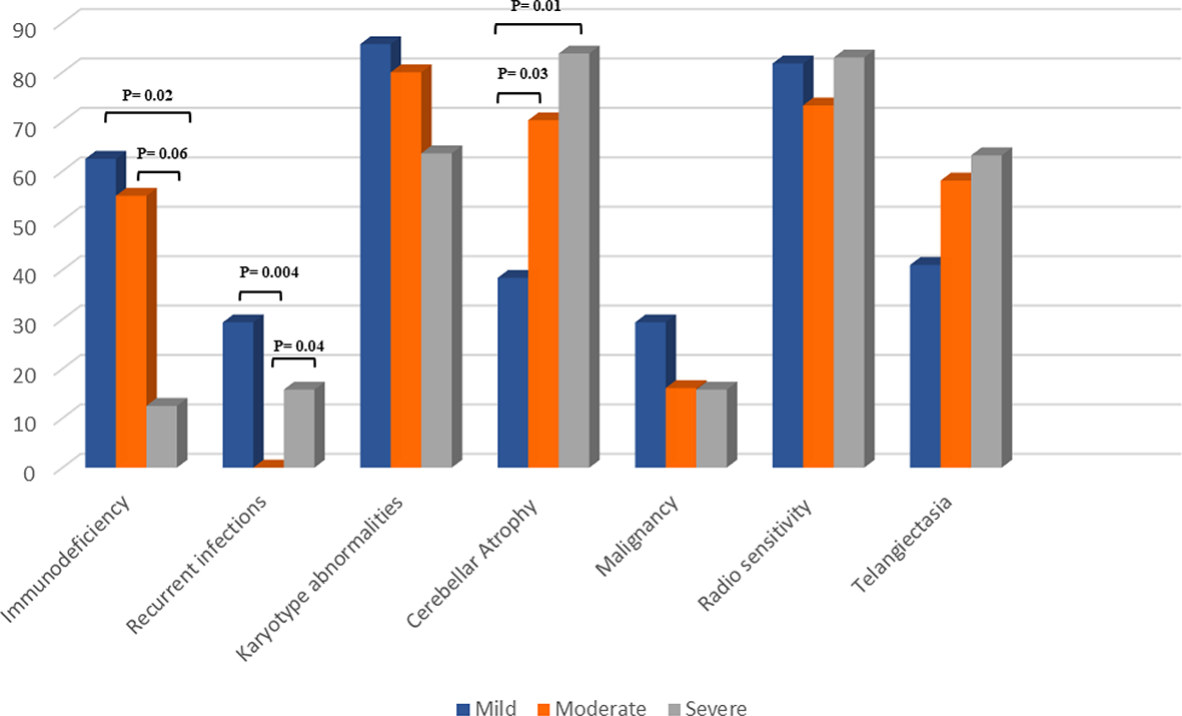
Comparison of clinical features of the 73 described variant AT patients in 3 groups.

Comparisons of clinical features of the 73 described variant atypical AT patients in 3 groups are illustrated in **Table 1** and **Figure 3**. Among 61 clinical recorded presentations, the most prevalent first presentation was neurologic symptoms in 56 (92%) of patients, with a median age of 10 (6 -17) years. Recurrent infections as the first presentation of the disease were reported in only 5 patients (8%) with a median (IQR) age of 1.5 (0 -7.5) years. Sixteen patients developed a malignancy (solid tumors, 53%; lymphoma, 40%) mostly in adulthood with a median (IQR) age of 37.5 (17 -55.5) years. Seven patients died owing to malignancy, as four cases (P44, P56, P27, P43) with solid tumors, two (P45, P53) due to leukemia, and one case (P11) resulting from lymphoma. The most common type of malignancy was lymphoma, leukemia and breast cancer. Autoimmunity and granulomatous lesion were observed in two patients (P73 and P1, respectively). Conjunctival telangiectasia was observed in 42 individuals with documented assessment (58%). The frequency of telangiectasia in the mild group (41%) was lower than the moderate (58%) and severe (63%) groups; however, the differences were not significant (*p*=0.18). In terms of the immunologic profile, 71% had IgA deficiency, followed by IgG subclass deficiency (19%) and hyper IgM phenotype (10%).

**Table 1:** Comparison of demographic, clinical, laboratory and molecular features in 3 groups.

Parameter	All patients	Group 1: Mild (- ataxia, -Wheelchair)	Group 2: Moderate (+ ataxia, -Wheelchair)	Group 3: Severe (+ ataxia, +Wheelchair)
Number of individuals	73	16	32	19
Male/Female	22/58	6/10	12/11	3/7
Presenting features	5 Recurrent infections 51 Neurologic problem	4 Recurrent infections 11 Neurologic problem	- Recurrent infections 22 Neurologic problem	- Recurrent infections 17 Neurologic problem
Median (IQR) age at onset, y	9(5.7-17)	11 (2.6-16.5)	17(6-29.5)	6.5 (2.3-9.7)
Median (IQR) age at onset of ataxia, y	13 (7.2-27.7)	–	27 (17-33)	8 (6.5-13)
Median (IQR) age at first wheelchair	18 (13.5-30)	–	–	18 (13.5-30)
Telangiectasia, N (%)	42 (58)	7 (41.2)	18 (58.1)	12 (63.2)
Immunodeficiency, N (%)	28 (40)	10 (62.5)	11 (55)	2 (12.5)
Recurrent infections, N (%)	10 (14)	5 (29.4)	–	3 (15.8)
Karyotype abnormalities, N (%)	33 (77)	6 (85.7)	16 (80)	7 (63.6)
Cerebellar Atrophy (MRI), N (%)	35 (65)	5 (38.4)	19 (70.3)	10 (83.8)
Malignancy, N (%)	12 (21)	5 (29.4)	5 (16.1)	3 (15.8)
Median (IQR) AFP (microgram/L)	120 (68-255)	118.5 (80.8-238.7)	115 (23.5-246.7)	127 (89.7-270.7)
Chromosomal radio-sensitivity, N (%)	26 (79)	9 (81.8)	11 (73.3)	5 (83)
Severity based on mutation, N (%)	58 (91) mild 6 (9) severe	12 (85.7) mild 2 (14.2) severe	27 (93.1) mild 2 (6.8) severe	15 (93.7) mild 1 (6.2) severe
Severity based on ATM activity, N (%)	32 (75) mild 11 (25) severe	7 (70) mild 3 (30) severe	16 (84.2) mild 3 (15.7) severe	7 (87.5) mild 1 (12.5) severe
Heredity (HMZ/HTZ), N (%)	21 (34)/40 (66)	7 (54)/6 (46)	7 (24)/22 (76)	6 (40)/9 (60)

HMZ; homozygous, HTZ; compound heterozygous, Y; year, AFP; alpha fetoprotein, IQR; interquartile range, MRI: magnetic resonance imaging, N; Count.

### Genetic Characteristics of Atypical AT Patients

Chromosomal radiosensitivity was normal in 7/33 individuals tested (21%), while 26/33 (79%) cases demonstrated chromosomal radiosensitivity. In 33 patients (77%), typical rearrangements of chromosomes 7 and 14 were demonstrated. Surprisingly, the frequency of abnormal karyotype in the mild group (85.7%) was slightly higher than other groups (moderate, 80%; severe, 63.6%); however, the differences were not significant (*p*=0.41).

**Figure 4:**
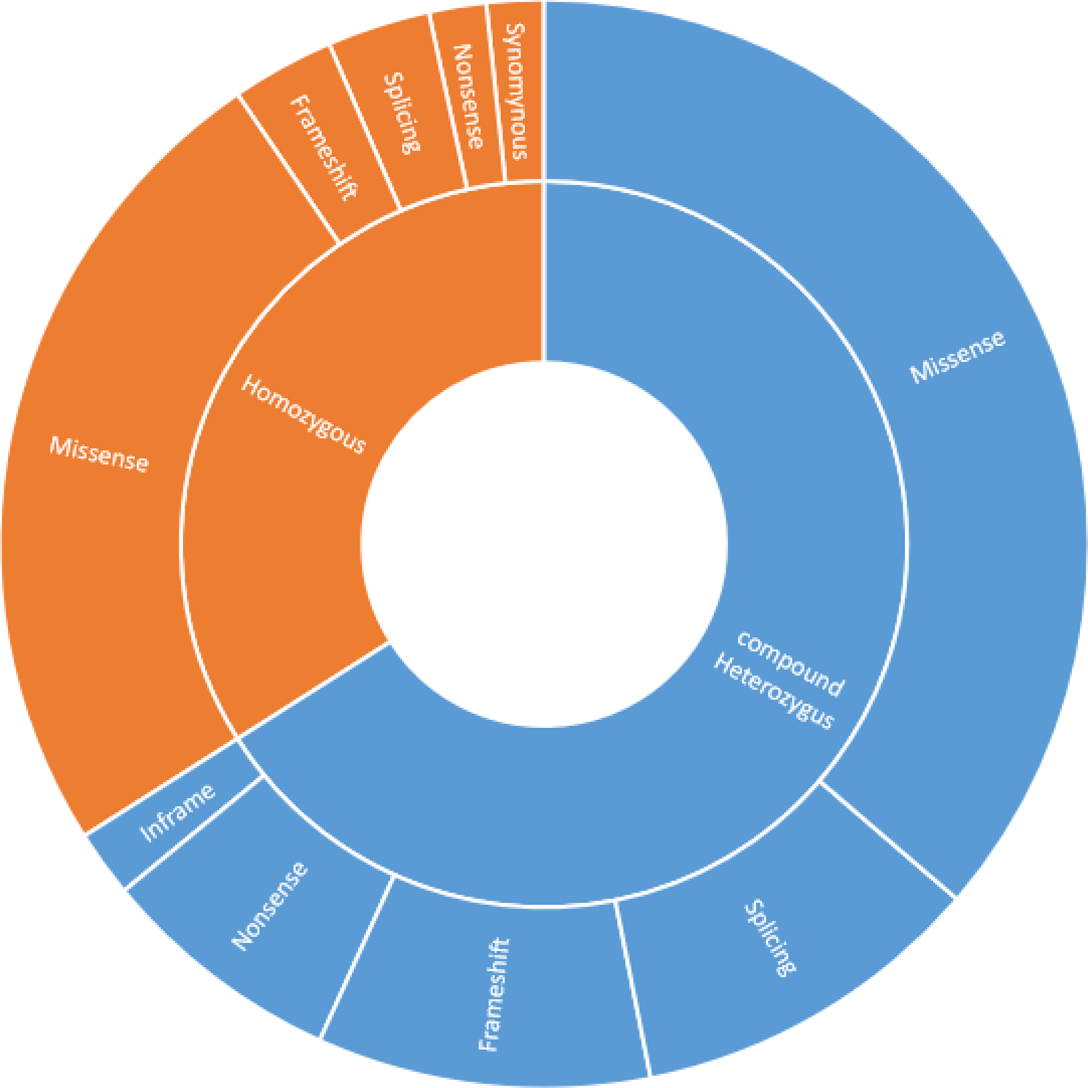
Comparison of genetics characteristics of the 61 described variant AT patients.

Genetic diagnosis based on *ATM* gene mutation was available for 61 patients and revealed that 40 out of 61 patients (66%) had a compound heterozygous mutation as well as homozygous mutations were found in 21 patients (34%). Missense mutations were the most prevalent, making up 60.6% of all mutations, followed by splice site (14%), frameshift (13%), and nonsense (9%) variants. Approximately 85% of patients carried at least 1 missense mutation. Severe mutations, including frameshift and/or nonsense for both alleles, were seen in only 6 (9%) patients; however mild alterations, including missense alterations for at least one allele, were documented in 58 (91%) patients. A comparison of genetic characteristics of the 61 described variant AT patients is provided in **Figure 4**.

### Functional ATM Analyses in Atypical AT Patients

**Figure 5:**
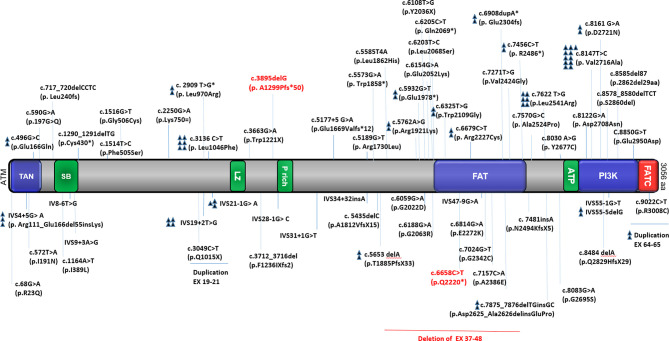
Distribution of known ATM mutations in different variant AT patients. The figure shows a schematic presentation of the ATM protein with the relative location of functionally important domains and the total mutations reported in the study (our 3 reported cases are indicated by red color). TAN; Tel1/ATM N-terminal motif, SB; substrate binding, LZ; leucine zipper, P rich proline-rich, FAT; FRAP-ATM-TRRAP domain, ATP; ATP binding site, PI3K; phosphatidylinositol 3-kinase-related kinase domain, FATC; FAT C-terminal domain (24–46).

ATM protein expression was detected in 36/44 atypical patients tested (82%) of whom 5 cases showed normal ATM expression. Residual ATM kinase activity was observed in 23/25 patients tested, all with missense or splice site (splice only in patients P54 and P55) mutations (**Supplementary Table 3**). Despite the mild mutations in some selected patients (P1, P22, and P23), no protein expression was observed. Moreover, in patients P26 (p.A2524P, p.W1221X) and P27 (p.R1730L and p.2862del29aa), despite low protein expression, no residual ATM kinase activity was detected. The distribution of known ATM mutations reported in the published study is depicted in **Figure 5**.

## Discussion

Here we reported 3 variant atypical Iranian AT cases (1.2% of total 249 AT patients registered in the national database of primary immunodeficiency) with late-onset of ataxia despite the presence of homozygous deleterious *ATM* mutation. Surprisingly, all of these cases presented autoimmune disorders. Moreover, for the first time, we comprehensively reviewed 73 reported variant AT patients with atypical late-onset ataxia in terms of clinical, immunological and genetic findings. In contrast to currently reported cases, we found that biallelic mild mutations mainly lead to an atypical phenotype with an increased risk of cancer (47). Our study confirms that disease severity in variant AT is milder compared to the classical form. Similar to what was observed in our cases, more than 70% of reported patients were not wheelchair-bound at the last examination, indicating unexpected slow progression in variant AT patients. Wheelchair use as a result of progressive cerebellar ataxia, presenting in early childhood, is a characteristic feature of classical AT patients (4, 48). This is consistent with our findings that even atypical patients with the severe phenotype (wheelchair-bound) demonstrated a significantly lower age of presenting onset and ataxia onset than other groups.

Previous reports revealed that within variant atypical AT patients, extrapyramidal movement disorders are dominating presentation rather than cerebellar signs. Extrapyramidal symptoms are commonly observed in predate ataxia in these patients, and the later occurring ataxia is of mild to moderate degree (7, 8). However, our study showed that in long follow-up they can occur relative to the same extent. On the other hand, Verhagen et al. (7) revealed that variant atypical AT patients generally presents with extrapyramidal symptoms such as chorea and resting tremor, respectively. We observed that tremor and chorea after ataxia were the most frequent presenting manifestation. Dystonia generally appears as a late manifestation and rarely develops as a presenting feature in AT patients (49). Incompatible with a report by Méneret et al. (50), in which dystonia was the presenting feature in 14% of variant patients, only 5% of reported variant atypical AT patients in our literature review presented with dystonia. As observed in our 3 cases, the majority of reported patients showed neurological abnormalities. However, two patients (P2 and P7) reported by Trimis et al. (51) and Soresina et al. (52) did not present neurological involvements but both had a history of telangiectasia, frequent respiratory infections, elevated serum IgM, and mild mutations. The absence of neurological features makes many difficulties in the diagnosis of the disease, especially in this type of variant, as patients might lead to being misdiagnosed as hyper IgM syndrome (HIgM). It has been postulated that other genetic modifiers or environmental factors can affect the neurological and clinical features of AT (52) as classical AT were observed in the close relatvies of two of our newly reported patients, most likely with the same *ATM* mutations. Cerebellar ataxia and extrapyramidal symptoms, similar to our 3 cases, were seen in most of the reported atypical patients indicating that these patients are vulnerable to both pathophysiologic mechanisms.

Dysarthria, as a most frequent cerebellar symptom (after ataxia), has been reported in 80% of AT patients (53); however, our study revealed a lower frequency of dysarthria in variant AT patients (Cases 2 and 3 of the present study and 64% of previously reported patients). In accordance with previous reports, the most common extrapyramidal symptoms were dystonia and tremor in reported variant AT (50). Carrillo et al. (54) reported that patients presenting predominant dystonia tend to have a later onset and a milder disease course. However, some studies observed the rare existence of dystonia among adolescents and adult AT patients (55). Nonetheless, it remains unclear at this time if the dystonic presentation is related to a specific genotypic abnormality (54). Chorea presented in 28% reviewed variant AT atypical patients; in contrast, few studies reported chorea mostly in mild patients (7). Méneret et al. (50), did not find chorea in any of their variant atypical patients, suggested that myoclonic dystonia in patients who were only assessed clinically can easily be mistaken for chorea. On the other hand, as severe chorea may cover the presence of ataxia, it should be considered that not all neurological signs indicate the cerebellum as the central lesion (49). Apraxia, as a hallmark of atypical AT, was appeared only in 25% of reported variant patients (7, 56). However, there is no direct correlation between the severity of eye movements and the severity of other neurologic symptoms (53).

In imaging of variant atypical AT patients, cerebellar morphology is often normal (5), though our study shows that only 35% reported atypical patients did not have cerebellar atrophy. However, a hypothesis suggested that some defects in abnormal ATM function may lead to cerebellar atrophy (57). Although multiple studies revealed that AT almost universally affects the cerebellum, the integrity of other mechanisms such as the anterior horn cells, basal ganglia, peripheral nerves, and dorsal columns may be influenced heterogeneously by the disease process (58, 59). Moreover, a significant increase in the frequency of cerebellar atrophy in the severe group demonstrate the relationship between the cerebellar atrophy and its effect on the walking ability of the patient; however, a relatively minor role of cerebellar cortical volume in the immediate expression of the neurodegeneration of AT has been reported (60).

Immunodeficiency with great variability is a characteristic feature of 60%–80% of AT patients (49); however, it is rarely observed in mild AT forms (7). As observed in the currently reported 3 cases, about 38% of individuals had an immunoglobulin deficiency, although most of them did not present considerable clinical features suggesting immune system dysfunction. It has been recognized that patients with ATM protein expression have an ameliorated immunological phenotype regardless of the presence or absence of residual kinase activity (8). On the other hand, patients with no kinase activity present significantly more severe immunological phenotypes than those with low levels of ATM activity (61). Besides, Van Os et al. (9) reported no correlation between the immune defects and expression of the ATM protein in AT. One possible mechanism of immunodeficiency has been proposed to small thymuses with poorly developed Hassal’s corpuscles in AT patients (62). Moreover, it has been reported that defective DNA repair and high levels of ROS production following dysfunction of ATM leads to the induction of apoptosis and cell death in ATM-deficient T cells (4). In terms of immunodeficiency, approximately 10% of AT patients exhibit the HIgM phenotype in which respiratory infections manifest at a very young age (63). Since neurological symptoms in these patients could be mild or absent, this could lead to misdiagnosis of variant AT with HIgM syndrome (64). Similar to cases 1 and 2 in the present study 10% of variant patients presented HIgM phenotype, as all were homozygous for a truncating mutation predicting the absence of ATM protein. The HIgM phenotype of AT is also associated with autoimmunity and cutaneous granulomatous disease (65–68). Except for case 3 of the present study, all reported patients with autoimmunity and cutaneous granuloma presented with HIgM phenotype along with splenomegaly. The etiology of these complications is not well-defined, but it seems that reduced regulatory T cells and increased ratios of CD21^−/low^ B cells, as well as disturbed naïve T and B cells along with splenomegaly, could be attributed to these patients (64–66). However, in our 3 evaluated cases, despite a decrease in naïve and regulatory T cells, nobody had an increase in CD21^−/low^ B cells. Our findings confirm our previous observation in which AT patients with class switch recombination (CSR) defect had lower CD21^−/low^ B cells compared to remaining (CSR Normal) patients (13). On the other hand, it has been reported that an abundance of CD21^−/low^ B cells in AT patients is associated with an increase of naïve B cell proliferation (69). Increased CD21^−/low^ B cells and its association with autoimmunity and splenomegaly have been reported by in common variable immunodeficiency (CVID) (70, 71), however there are no other reports in AT patients to show an increase of this subset in autoimmune disease.

Rearrangements of chromosomes 7 and 14 presented in most reported variant AT patients in this study. Interestingly, the same was true for all reported variant AT patients in the literature (72). On the other hand, it has been postulated that chromosomal translocation between chromosomes 7 and 14 that are associated with Ig/TCR genes may be involved in Immunodeficiency in AT patients (73). This is confirming our data that the frequency of immunodeficiency and karyotype abnormalities are relatively associated among the groups. Also, by comparison, the frequency of karyotype abnormalities and immunodeficiency between our 3 groups, we demonstrated the inverse relationship between the frequency of them with the severity of neurological phenotypes, which indicates the severity of neurological phenotypes are not associated with the occurrence of karyotype abnormalities and immunodeficiency.

Among AT patients, the lifetime risk of malignancy is 10%–38% (74, 75). Furthermore, Verhagen et al. reported that cancer can occur later in the life of variant AT, which typically consists of solid malignancies rather than lymphoid cancer (7, 8). In line with the literature, 21% reported atypical patients who suffered from malignancies presented mostly in adulthood with predominately solid tumors. We found that about 85% of individuals with malignancy had a missense mutation, which can cause the production of a mutant ATM protein with retained kinase activity. Our findings correspond with the results of Schon et al. (47), indicating variant patients with a missense mutation are almost fivefold more at-risk to develop a malignancy. Moreover, previous reports revealed p.Ala2067Asp and p.Val2424Gly missense mutations as a high-risk cause of malignancy (76, 77). The nature of the underlying *ATM* mutation, which on the one hand does not protect against cancer development or radiation damage, but on the other, allows ATM expression with sufficient activity to protect against severe neurodegeneration, has not been fully understood. Maybe modifying genes/regulators affect these clinical and cellular responses (78). Infection and malignancy are the main causes of mortality in patients with AT (9). Our report revealed that malignancies, instead of respiratory failure, were the main cause of death in variant AT patients, which is similar to previous reports (8).

Consistent with the expected notion, we found that more than 80% of individuals had a missense mutation, possibly causing milder neurological features (7, 50). Although the correlation of neurodegenerative phenotype and defect of ATM is unclear, the hypothesis suggested that ATM protein expression may allow neurons to initiate apoptosis pathways or repair damaged DNA (79, 80). On the other hand, Verhang et al. (7) suggested that in AT variant, a better-conserved function of the mitochondrion due to less oxidative stress as a consequence of residual ATM kinase activity can describe the milder clinical symptoms. They also suggested that some splicing mutations with less ATM protein expression and kinase activity may result in a more severe phenotype rather than missense mutation. Besides, some cases (P26 and P27) showed no ATM kinase activity associated with the expressed protein despite ATM missense mutations and reduced ATM protein expression. A more sensitive assay would help detect low differential levels of ATM activity associated with different ATM proteins, while there may be some activity retained to cause milder features of the disease (47). Another possible explanation is that missense mutation in the kinase domain, may lead to a normal level of protein expression but not functional (kinase-dead) protein (81).

Here, we show that similar to our 3 cases, approximately 9% of variant AT patients with mild clinical phenotype can appear with the presence of null *ATM* mutations (frameshift and nonsense mutations causing truncation), leading to an absence of ATM protein and no kinase activity. These observations raise the possibility that the neuronal cells of these patients are affected by a modifying gene leading to a compensatory pathway that protects against the consequences of ATM loss (57). The rarity of such patients probably indicates that the highly influential alleles of such modifier genes are rare (60). Rarely, patients with mild or classical AT show a normal intensity of the ATM band on a Western blot (82, 83); as our study revealed that 9% of variant AT patients showed a normal ATM protein expression. One of the limitations of our study is that we did not have extra access to the material to check the level of ATM protein as well as its activity in our patients. Our previous report also showed that approximately 90% of AT patients with classical presentations had a homozygous mutation (84); however, we found that more than 60% of the reported variant AT patients had a compound heterozygous mutation. However, we did not find a significant genotype-phenotype association with regards to neurological groups in the study overall, indicating an additional influence of shared environmental factors or disease-modifying genes. Future examination of newly identified ATM functions may provide vital insights into the relationship between mutation and phenotype (10, 85–89).

## Conclusion

There is no universally accepted definition of the variant AT phenotype. The clinical spectrum of these atypically affected AT patients may be similar, although the underlying causative *ATM* mutations may be different. Our results support previous results that AT is not only due to pure ataxia and may appear as only different extrapyramidal signs or even normal neurological symptoms. As they are likely to be under or misdiagnosed, early diagnosis using newborn screening or unbiased next-generation sequencing on suspected individuals is crucial considering the increased risk of malignancies, the related higher risk for side effects of radiation and certain chemotherapy regimens in cancer treatment, genetic counseling, carrier detection within the affected family, and prenatal diagnosis.

## Data Availability Statement

The original contributions presented in the study are included in the article/**Supplementary Material**. Further inquiries can be directed to the corresponding authors.

## Ethics Statement

Patients signed informed consent regarding publishing their data and photographs.

## Authors Contributions

TM, AA, and RY designed and wrote the manuscript. SD, MH, SA, and MS-S collected clinical data and provided samples of patients for genetic and laboratory analyses. PA, NR, and HA performed and analyzed the results of next-generation sequencing and Sanger sequencing. TM performed and analyzed immunological tests of patients. All authors reviewed, contributed, and approved the final draft of the manuscript.

## Funding

This work was supported by grants provided by the Jeffrey Modell Foundation, Åke Wibergs stiftelse and Crafoord Foundation.

## Conflict of Interest

The authors declare that the research was conducted in the absence of any commercial or financial relationships that could be construed as a potential conflict of interest.

## Publisher’s Note

All claims expressed in this article are solely those of the authors and do not necessarily represent those of their affiliated organizations, or those of the publisher, the editors and the reviewers. Any product that may be evaluated in this article, or claim that may be made by its manufacturer, is not guaranteed or endorsed by the publisher.

## Supplementary Material

Supplementary Data Sheet Atypical Ataxia Presentation in Variant Ataxia Telangiectasia: Iranian Case-Series and Review of the Literature. Tannaz Moeini Shad et al.

Supplementary Figure 1 Flowchart of the systematic search and study selection process.

Supplementary Table 1 Demographic, clinical, laboratory and molecular features of three atypical AT cases. ITP; idiopathic thrombocytopenic purpura, AFP; alpha-fetoprotein, WBC; white blood cells, IVIG; intravenous immunoglobulin

Supplementary Table 2 Demographic, clinical and laboratory characteristics of the 73 described variant atypical AT patients. N, number; P, patient; M, male, F, female; Y, year; AFP, alpha-fetoprotein; ITP, idiopathic thrombocytopenic purpura; IVIG, intravenous immunoglobulin; IVCY, high-dose intravenous cyclophosphamide; L, low; H, high; N, normal; NR, not reported.

Supplementary Table 3 Genetics characteristics of the 73 described variant atypical AT patients. ATM, ataxia‐telangiectasia mutated; HMZ, homozygous; HTZ, compound heterozygous; NR, not reported. *Based on Western blot experiment. **Based the presence or absence of protein patients were divided to two group of severe (without residual of expression or function) and mild (with residual of expression and function.
