# Supplementary material for: Atypical Ataxia Presentation in Variant Ataxia Telangiectasia: Iranian Case-Series and Review of the Literature
Source: Front Immunol. 2022 Jan 14;12:779502. doi: 10.3389/fimmu.2021.779502 (PMC8795590; doi:10.3389/fimmu.2021.779502)
Supplement: Supplementary Data Sheet — Atypical Ataxia Presentation in Variant Ataxia Telangiectasia: Iranian Case-Series and Review of the Literature. Tannaz Moeini Shad et al. [file DataSheet_1.pdf]

Supplementary data

Atypical Ataxia Presentation in Variant Ataxia Telangiectasia: Iranian Case-Series and Review of the Literature

Tannaz Moeini Shad et al.

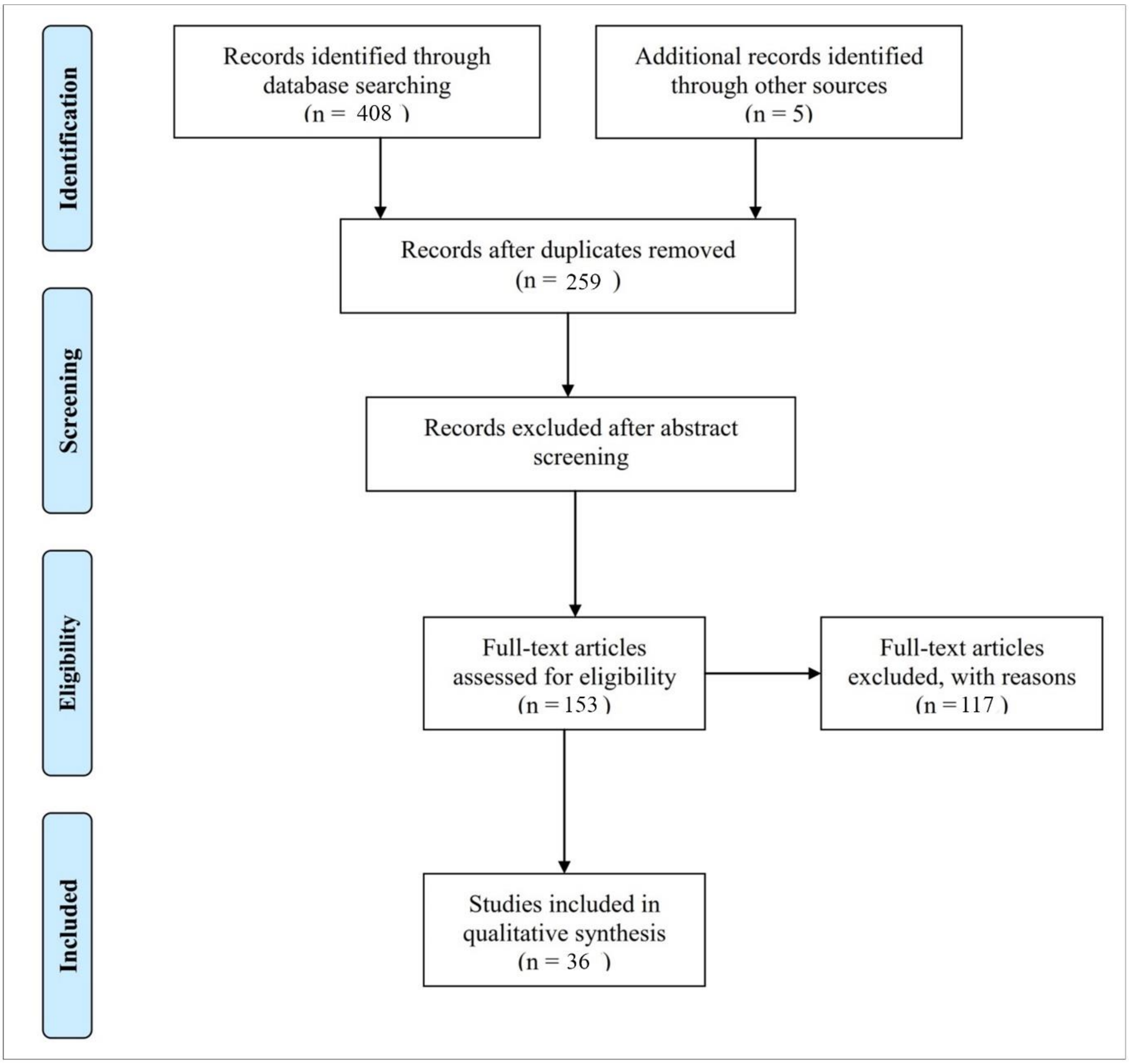

Supplementary Figure 1. Flowchart of the systematic search and study selection process.

Supplementary Table 1. Demographic, clinical, laboratory and molecular features of three atypical AT cases.

Parameters	Case 1	Case 2	Case 3	Normal values
**Sex**	M	F	F	
**Age, y**	9	13	24	
**Presenting features, y**	respiratory infections (0.7 y)	thrombocytopenia, purpura (3 y)	ataxic gait, telangiectasia (8 y)	
**Age at onset of ataxia, y**	6	6	8	
**Telangiectasia**	+	+	+	
**Chromosomal instability**	+	+	+	
**Autoimmunity (Treatment)**	ITP (IVIG, corticosteroids)	ITP (Prednisolone)	Psoriasis (Adalimumab)	
**Recurrent infections**	+	+	-	
**WBC × 10^3 (cell/ µL)**	2.7	10.6		4.5–13.5
**Hemoglobin (g/dL)**	11.2	11.9		11.5–15.5
**Anti-Diphtheria (IgG; IU/mL)**	<0.1	0.8		>0.1
**Anti-Tetanus (IgG; IU/mL)**	<0.1	0.2		>0.1
**AFP (ng/mL)**	275.5 ↑	152 ↑	411 ↑	<8.5
**IgG (mg/dL)**	27 ↓	227 ↓	1113	500-1600
**IgA (mg/dL)**	2 ↓	3 ↓	8 ↓	33-202
**IgM (mg/dL)**	724 ↑	3021 ↑	225	38-251
**Immunoglobulin profile**	HIgM	HIgM	IgA deficient	
**CD8 + T cells, (cell/µL)**	90 ↓	28 ↓	420	258-797
**CD8 + Naïve T cells, (cell/µL)**	1.9 ↓	3.7 ↓	54.2 ↓	105-476
**CD8 + Central Memory T cells, (cell/µL)**	0.4 ↓	0.1 ↓	24.3	1-55
**CD8 + TEMRA T cells, (cell/µL)**	46 ↓	14 ↓	148	56-282
**CD8 + Effector memory T cells, (cell/µL)**	42	10 ↓	194	28-324
**CD4 + T cells, (cell/µL)**	56 ↓	111 ↓	633	512-1253
**CD4 + Naïve T cells, (cell/µL)**	1.7 ↓	98 ↓	50 ↓	179-827
**CD4 + Central Memory T cells, (cell/µL)**	5 ↓	3 ↓	277 ↑	67-163
**CD4 + TEMRA T cells, (cell/µL)**	4.8 ↓	1.1 ↓	33.8	7-117
**CD4 + Effector memory T cells, (cell/µL)**	44 ↓	9 ↓	273	58-577
**CD4 + regulatory T cells, (cell/µL)**	2.4 ↓	3.6 ↓	2 ↓	5-25.5
**CD19+ B cells, (cell/µL)**	24.4 ↓	2 ↓	250.6	88.5-284.5
**Naïve B cells, (cell/µL)**	12.1 ↓	0 ↓	68.9	36-227
**Marginal zone B cells, (cell/µL)**	4.4	0 ↓	22.6 ↑	1.5-21
**Switched Memory B cells, (cell/µL)**	0.7 ↓	0.2 ↓	6	2.5-22
**IgM-only memory B cells, (cell/µL)**	1.22	0 ↓	17.54	0.2-26
**Transitional B cells, (cell/µL)**	0.33 ↓	0.02 ↓	6.56	2.5-41.5
**$CD21^{low}$ B cells, (cell/µL)**	0.2	0 ↓	7.5	0.1-14
**Plasmablast, (cell/µL)**	0.37 ↓	1.09	1.5	0.4-9.5
***ATM* mutations**	c.3895 delG	Large deletion EX37-48	c.6658C>T	-
**Mutation Type**	Frameshift	Large deletion	Nonsense	-
**Severity based on mutation**	Severe	Severe	Severe	-
**Predicted Protein Alternation**	p.A1299Pfs*50	-	p.Q2220*	-

**ClinVar reported**	rs786203501/ PP5	Not reported	rs1060501536/ PP5	-
**gnomAD population frequency**	PM2	PM2	PM2	
**ACMG criteria**	PVS1	PVS1	PVS1	-
**Heredity**	Homozygous	Homozygous	Homozygous	-
ITP; idiopathic thrombocytopenic purpura, AFP; alpha-fetoprotein, WBC; white blood cells, IVIG; intravenous immunoglobulin, HIgM: Hyper IgM phenotype.				

Supplementary Table 2. Demographic, clinical and laboratory characteristics of the 73 described variant atypical AT patients.

No	Sex/ Origin	First presenting features	IgG	IgA	IgM	AFP	Age at onset of ataxia, y	Wheelchair	Telangiectasia	Cerebellar Atrophy (MRI)	Chromosomal instability	Karyotype abnormalities	Major symptoms	Ref.
**Group I (Mild): - no ataxic signs**														
**P1**	F/ Iraq	Recurrent infections (3 y)	L	N	H	H	no ataxia	no	+	+	+		Otitis media, sinusitis, pneumonia, cutaneous granulomas, café au lait macule	(68)
**P2**	F/ Italy	Recurrent respiratory infections (0.1 y)	L	N	H	H	no ataxia	no	+		+		Recurrent respiratory infections	(30)
**P4**	M/ Circassia	Neurologic problem (9.5 y)	N	N	N	H	no ataxia	no	+	+	+			(38)
**P7**	F/ Greece	Bronchitis (0.1 y)	N	L	H	H	no ataxia	no	+	+	+		Recurrent nasal infections, bronchitis	(29)
**P10**	F/ Belgium	Gait unsteadiness		L	N	H	no ataxia	no	-	-	+	-	Recurrent sinopulmonary	(3)
**P20**	M/ Netherlands	Tremor (18 y)	N	N	H	H	no ataxia	no	+	+	-	+		(69)
**P21**	M/ Netherlands	Tremor (26 y)	N	N	N	H	no ataxia	no	+	-	+	+		(69)
**P25**	F/ Arab	Mild myoclonus	N	L	N	H	no ataxia	no	-		+			(70)
**P29**	F/ Germany	Neurologic problem (childhood)	N	L	N	H	no ataxia	no	-	-				(71)
**P31**	M/ Netherlands	Hypotonia, clumsiness, slurred speech (2.5 y)	N	N	N	H	no ataxia	no	-		+		T-cell lymphoblastic non-Hodgkin lymphoma (12y)	(72)
**P48**	M/ USA	Cough, facial swelling (17 y)	N	N	N	H	no ataxia	no	-	-		+	T-cell acute lymphoblastic leukemia (17 y)	(73)
**P49**	F/ Germany	Neurologic problem (12 y)	N	N	N	H	no ataxia	no	-	-			Pneumonia, recurrent bronchitis, pharyngitis,	(74)
**P50**	F/ India	Dystonia (15 y)				H	no ataxia	no	+	-	-	+	Orofacial dyskinesias	(32)
**P59**	F/ Netherlands	Resting tremor and dysarthria (12 y)	N	N	N	H	no ataxia	no	-	-			Breast cancer (32 y)	(7)
**P64**	F/ -		N	L	N	H	no ataxia	no	-	-		+		(75)

**P65**	-/ France		N	L	N	H	no ataxia	no	-				T-lymphoma lymphoblastic (4 y), Gliomatosis cerebri (13 y)	(76)
**Group II (Moderate): + ataxic signs, - not using a wheelchair**														
**P3**	M/ Circassia	Gait unsteadiness, dysarthria (6 y)	N	L	H	H	6	no	+	+	+			(38)
**P8**	F/ UK	Ataxia (52 y)	L	N	L	H	52	no (walk with walking aids)	+	+	+			(77)
**P9**	F/ UK	Chorea (teenage)	N	L	N	H	teenage	no	+	+	+	+		(35)
**P11**	M/ Netherlands	Neurologic problem (10 y)	N	L	N	H	>11	no	-	+	+	+	died from lymphoma (57 y)	(78)
**P12**	F/ Italy	Ataxia (27 y)	N	N	N	H	27	no	-	-	+	+		(34)
**P13**	M/ UK	Ataxia, telangiectasia (17 y)	N	N	N		17	no	+	+	+			(37)
**P14**	M/ UK	Ataxia (22 y)	N	N	N		22	no	+	+	+			(37)
**P15**	M/ -					H	29	no	+	-	-	+		(79)
**P16**	M/ -					H	30	no	+	+	+	+		(79)
**P17**	M/ -					H	37	no	+			+		(79)
**P18**	F/ -					H	32	no	+	-		+		(79)
**P23**	M/ Italy	Gait unsteadiness (35 y)	N	N	N	H	39	no (walk with leg orthoses)	+	+	-			(80)
**P24**	M/ -	Neurologic problem (9 y)	N	L	N	H	9	no	+					(81)
**P32**	F/ India		N	N	H	H	28	no			+	+		(82)
**P38**	NR	Dysarthria (6 y)	N	N	N	H	>6	no	-	+		-		(28)
**P39**	NR	Dysarthria (6 y)	N	L	N	H	>6	no	-	+		-		(28)
**P40**	NR	Dystonia (8 y)	N	N	N	H	>8	no	-	+		-		(28)
**P41**	NR	Dystonia (6 y)	N	N	N	H	>6	no	-	+		-		(28)
**P44**	F/ Japan	Tremor, gait unsteadiness (17 y)	N	N	N	H	17	no	-	+			died from gallbladder cancer (70 y)	(83)

**P45**	F/ Japan	Tremor, gait unsteadiness (17 y)	N	N	N	H	17	no	-	+			died from chronic lymphocytic leukemia (76 y)	(83)
**P47**	F/ Austria	Gait unsteadiness (34 y)	N	N	N	N	34	no	+	+				(84)
**P51**	M/ Netherlands	Resting tremor (26 y)	N	N	N	H	>6	no	+	+	+	+		(7)
**P52**	M/ Netherlands	Resting tremor (16 y)	N	N	N	H	>6	no	+	-	-	+		(7)
**P53**	M/ Netherlands	Resting tremor (34 y)	N	N	N	H	>6	no	+	-		+	died from acute lymphoblastic leukemia (51 y)	(7)
**P56**	M/ Netherlands	Chorea-athetosis (0.5 y)	N	N	N	H	>6	no	-	-		+	died from an ectopic pituitary tumor (23 y)	(7)
**P57**	F/ Netherlands	Chorea-athetosis (0.5 y)	N	N	N	H	>6	no	-	-	-	+		(7)
**P58**	F/ Netherlands	Chorea-athetosis (4 y)	N	N	N	H	>6	no	-	+	+	+		(7)
**P62**	F/ Netherlands	Gait disturbance and resting tremor (32 y)	N	N	N	H	>6	no	+	-	+	+		(7)
**P66**	-/ France		N	L	N	H	>15	no	-					(76)
**P67**	-/ France		N	N	N	H	>7	no	+	+			Dermatofibrosarcoma (17 y)	(76)
**P69**	-/ France		N	N	N	H	26	no	+	+		+		(76)
**P71**	-/ France		N	N	N	H	15	no	-	+		+	Carcinoma thyroid	(76)
**Group III (Severe): + ataxic signs, + using a wheelchair**														
**P5**	M/ Turkey	Gait unsteadiness (7 y)	L	N	N	H	7	(13 y)	+				Pneumonia	(85)
**P6**	F/ Brazil	Ataxia (6 y)	N	L	N	H	6	yes	+				Pneumonia, died from severe pulmonary infection (16 y)	(86)
**P22**	F/ Italy	Tremor (13 y)	N	N	N	H	28	35	+	+	+			(80)
**P30**	M/ -	Progressive motor disturbances (6 y)	N	N	N	H	6	(18 y)	-	+	+	+		(87)
**P33**	NR	Ataxia (9 y)	N	N	N	H	9	(14 y)	+	+		+		(28)
**P34**	NR	Ataxia (7 y)	N	N	N	H	7	(11 y)	+			-		(28)
**P35**	NR	Ataxia (8 y)	N	N	N	H	8	(14 y)	+	+		-		(28)
**P36**	NR	Ataxia (5 y)	N	N	N	N	5	(12 y)	-			-		(28)
**P37**	NR	Ataxia (14 y)	N	N	N	H	14	(25 y)	-	+		-		(28)
**P42**	NR	Ataxia (10 y)	N	N	N	H	10	(18 y)	+	+		+		(28)

**P46**	F/ Saudi	Gait unsteadiness (14 y)	N	N	N	H	14	(22 y)	+	+				(88)
**P54**	Netherlands	Chorea-athetosis (1 y)	N	N	N	H	>6	(> 15 y)	-	+	-			(7)
**P55**	F/ Netherlands	Chorea-athetosis (1 y)	N	N	N	H	>6	(> 15 y)	+	+	+	+		(7)
**P60**	F/ Netherlands	Chorea-athetosis (0.5 y)	N	N	N	H	>6	(> 15 y)	+	-			Breast cancer (32 y)	(7)
**P61**	F/ Netherlands	Chorea-athetosis (1.5 y)	N	N	N	H	>6	(> 15 y)	-	-				(7)
**P63**	F/ Netherlands	Distal muscle weakness (6 y)	N	N	N	H	>6	(> 15 y)	+	+	+			(7)
**P68**	-/ France		N	N	N	H	12	(23 y)	+	+		+		(76)
**P70**	-/ France		N	N	N	H	<50	(50 y)	-			+		(76)
**P72**	M/ Germany	Ataxia (7y)					7	(45 y)	-		+	+	Recurrent infections, died from cachexia (60 y)	(50)
**Data on using a wheelchair not available (Not included in the classification)**														
**P19**	F/ Italy	Neurologic problem				H	9		+	-	+	+		(89)
**P26**	-/ France		N	L	N	N	6		+			+		(63)
**P27**	F/ France		N	L	N	H	8		+		+	+	died from breast cancer (43 y)	(63)
**P28**	-/ France		N	N	N	H	10		-			+		(63)
**P43**	-/ Netherlands	The neurologic problem, recurrent respiratory infections (9 y)	L	L	H	H	9		+				Recurrent infections, died from hepatocellular carcinoma (10 y)	(41)
**P73**	M/ Japan	Upper respiratory tract infections, gastrointestinal tract infections, pharyngitis (3 y)	L	L	H		6		+	+			ITP (3y), Treatment: (IVIG, Prednisolone, IVCY, cyclophosphamide), splenomegaly	(90)

N; number, P; patient, M; male; F; female, Y; year, AFP; alpha-fetoprotein, ITP: idiopathic thrombocytopenic purpura, IVIG: intravenous immunoglobulin, IVCY: high-dose intravenous cyclophosphamide, L: low, H: high, N: normal, NR: not reported.

Supplementary Table 3. Genetics characteristics of the 73 described variant atypical AT patients.

<table>
<tr><th>N</th><th>Gene</th><th>Mutation at cDNA level</th><th>Mutation at protein level</th><th>HEREDITY</th><th>Mutation Type</th><th>Severity based on mutation</th><th>ATM protein/ATM kinase activity *</th><th>Severity based on functional assay**</th></tr>
<tr><td colspan="9">Group I (Mild): - no ataxic signs</td></tr>
<tr><td>P1</td><td>ATM</td><td>c.2250G>A</td><td>p.Lys750=</td><td>HMZ</td><td>Synonymous</td><td>Mild</td><td>Absence of ATM protein</td><td>Severe</td></tr>
<tr><td>P2</td><td>ATM</td><td>c.6679C>T
c.8484delA</td><td>p. Arg2227Cys
p.Gln2828fs*</td><td>HTZ</td><td>Missense,
Frameshift</td><td>Mild</td><td>Reduction of ATM protein</td><td>Mild</td></tr>
<tr><td>P4</td><td>ATM</td><td>c.5653delA</td><td>p.Thr1885fs*</td><td>HMZ</td><td>Frameshift</td><td>Severe</td><td>Absence of ATM</td><td>Severe</td></tr>
<tr><td>P7</td><td>ATM</td><td>c.8850G>T</td><td>p.Glu2950Asp</td><td>HMZ</td><td>Missense</td><td>Mild</td><td>NR</td><td>NR</td></tr>
<tr><td>P10</td><td>ATM</td><td>c.8122G>A
c.8851-1G>T</td><td>p. Asp2708Asn
-</td><td>HTZ</td><td>Missense,
splicing</td><td>Mild</td><td>Reduction of ATM protein with kinase activity</td><td>Mild</td></tr>
<tr><td>P20</td><td>NR</td><td>NR</td><td>NR</td><td>NR</td><td>NR</td><td>NR</td><td>NR</td><td>NR</td></tr>
<tr><td>P21</td><td>NR</td><td>NR</td><td>NR</td><td>NR</td><td>NR</td><td>NR</td><td>NR</td><td>NR</td></tr>
<tr><td>P25</td><td>ATM</td><td>c.1514T>C</td><td>p.Phe505Ser</td><td>HMZ</td><td>Missense</td><td>Mild</td><td>Reduction of ATM protein with kinase activity</td><td>Mild</td></tr>
<tr><td>P29</td><td>ATM</td><td>c.8147T>C
c.8578_8580delTCT</td><td>p. Val2716Ala
p.S2860del</td><td>HTZ</td><td>Missense,
In- Frame</td><td>Mild</td><td>NR</td><td>NR</td></tr>
<tr><td>P31</td><td>ATM</td><td>c.5932G>T</td><td>p.Glu1978*</td><td>HMZ</td><td>Nonsense</td><td>Severe</td><td>Absence of ATM protein</td><td>Severe</td></tr>
<tr><td>P48</td><td>ATM</td><td>c.5585T>A</td><td>p.Leu1862His</td><td>HMZ</td><td>Missense</td><td>Mild</td><td>Reduction of ATM protein</td><td>Mild</td></tr>
<tr><td>P49</td><td>ATM</td><td>c.5573G>A
c.6154G>A</td><td>p. Trp1858*
p.Glu2052Lys</td><td>HTZ</td><td>Nonsense,
Missense</td><td>Mild</td><td>NR</td><td>NR</td></tr>
<tr><td>P50</td><td>ATM</td><td>c.590G>A</td><td>p. Gly197Gln</td><td>HMZ</td><td>Missense</td><td>Mild</td><td>Reduction of ATM protein with kinase activity</td><td>Mild</td></tr>
<tr><td>P59</td><td>ATM</td><td>c.8147 T>C
Unidentified</td><td>p. Val2716Ala
Unidentified</td><td>?</td><td>Missense
-</td><td>Mild</td><td>NR</td><td>NR</td></tr>
<tr><td>P64</td><td>ATM</td><td>c.572T>A
c.6679C>T</td><td>p.Ile191Asn
p.Arg2227Cys</td><td>HTZ</td><td>Missense,
Missense</td><td>Mild</td><td>Reduction of ATM protein with kinase activity</td><td>Mild</td></tr>
<tr><td>P65</td><td>ATM</td><td>c.4776+1G>T
c.6814G>A</td><td>-
p.Glu2272Lys</td><td>HTZ</td><td>Splicing,
Missense</td><td>Mild</td><td>Reduction of ATM protein with kinase activity</td><td>Mild</td></tr>
<tr><td colspan="9">Group II (Moderate): + ataxic signs, - not using a wheelchair</td></tr>
</table>

**P3**	*ATM*	c.5653delA	p.Thr1885fs*	HMZ	Frameshift	Severe	Absence of ATM protein	Severe
**P8**	*ATM*	c.1066-6T>G<br>c.7271T>G	-<br>p.Val2424Gly	HTZ	Splicing,<br>Missense	Mild	NR	NR
**P9**	*ATM*	c.5177þ5 G>A<br>c.1290_1291delTG (	p.Glu1669Valfs*12<br>p.Cys430*	HTZ	Frameshift,<br>Nonsense	Severe	Absence of ATM protein	Severe
**P11**	NR	NR	NR	NR	NR	NR	NR	NR
**P12**	*ATM*	c.8030 A>G<br>c.7481insA	p. Tyr2677Cys<br>p.Asn2494Lysfs*	HTZ	Missense,<br>Frameshift	Mild	Reduction of ATM protein with kinase activity	Mild
**P13**	*ATM*	c.5762A>G	p.Arg1921Lys	HMZ	Missense	Mild	Reduction of ATM protein with kinase activity	Mild
**P14**	*ATM*	c.5762A>G	p.Arg1921Lys	HMZ	Missense	Mild	Reduction of ATM protein with kinase activity	Mild
**P15**	*ATM*	c.7622T>G<br>c.3136C>T	p.Leu2541Arg<br>p.Leu1046Phe	HTZ	Missense,<br>Missense	Mild	Reduction of ATM protein with kinase activity	Mild
**P16**	*ATM*	c.7622T>G<br>c.3136C>T	p.Leu2541Arg<br>p.Leu1046Phe	HTZ	Missense,<br>Missense	Mild	Reduction of ATM protein with kinase activity	Mild
**P17**	*ATM*	c.7622T>G<br>c.3136C>T	p.Leu2541Arg<br>p.Leu1046Phe	HTZ	Missense,<br>Missense	Mild	Reduction of ATM protein with kinase activity	Mild
**P18**	*ATM*	c.IVS21-1G> A<br>c.8147T>C	-<br>p.Val2716Ala	HTZ	Missense,<br>Missense	Mild	Reduction of ATM protein with kinase activity	Mild
**P23**	*ATM*	c.6325T>G	p.Trp2109Gly	HMZ	Missense	Mild	Absence of ATM protein	Severe
**P24**	NR	NR	NR	NR	NR	NR	NR	NR
**P32**	NR	NR	NR	NR	NR	NR	NR	NR
**P38**	*ATM*	IVS19+2T>G<br>c.8147T>C	-<br>p.Val2716Ala	HTZ	Splicing,<br>Missense	Mild	NR	NR
**P39**	*ATM*	IVS19+2T>G<br>c.8147T>C	-<br>p.Val2716Ala	HTZ	Splicing,<br>Missense	Mild	NR	NR
**P40**	*ATM*	IVS19+2T>G<br>c.8147T>C	-<br>p.Val2716Ala	HTZ	Splicing,<br>Missense	Mild	NR	NR
**P41**	*ATM*	IVS19+2T>G<br>c.8147T>C	-<br>p.Val2716Ala	HTZ	Splicing,<br>Missense	Mild	NR	NR
**P44**	*ATM*	c.496G>C	p.Glu166Gln	HMZ	Missense	Mild	NR	NR

**P45**	*ATM*	c.496G>C	p.Glu166Gln	HMZ	Missense	Mild	NR	NR
**P47**	*ATM*	c.6205C>T<br>c.1235+3A>G	p. Gln2069*<br>-	HTZ	Nonsense,<br>splicing	Mild	NR	NR
**P51**	*ATM*	c.3136 C>T<br>c.7622 T>G	p. Leu1046Phe<br>p.Leu2541Arg	HTZ	Missense,<br>Missense	Mild	Reduction of ATM kinase activity	Mild
**P52**	*ATM*	c.3136 C>T<br>c.7622 T>G	p. Leu1046Phe<br>p.Leu2541Arg	HTZ	Missense,<br>Missense	Mild	Reduction of ATM kinase activity	Mild
**P53**	*ATM*	c.3136 C>T<br>c.7622 T>G	p. Leu1046Phe<br>p.Leu2541Arg	HTZ	Missense,<br>Missense	Mild	NR	NR
**P56**	*ATM*	c. 2909 T>G<br>c.6908dupA	p. Leu970Arg<br>p.Glu2304fs*	HTZ	Missense,<br>Frameshift	Mild	NR	NR
**P57**	*ATM*	c. 2909 T>G<br>c.6908dupA	p. Leu970Arg<br>p.Glu2304fs*	HTZ	Missense,<br>Frameshift	Mild	Normal ATM protein expression	Mild
**P58**	*ATM*	c.5932 G>T<br>c.8147 T>C	p. Glu1978*<br>p.Val2716Ala	HTZ	Nonsense,<br>Missense	Mild	Normal ATM protein expression	Mild
**P62**	*ATM*	c.2922–1G>A<br>c.8147 T>C	-<br>p.Val2716Ala	HTZ	Splicing,<br>Missense	Mild	Reduction of ATM kinase activity	Mild
**P66**	*ATM*	c.7157C>A	p.Ala2386Glu	HMZ	Missense	Mild	Reduction of ATM protein with kinase activity	Mild
**P67**	*ATM*	c.3712_3716del<br>c.1164A>T	p.Leu1238fs*<br>p.Ser49Phe	HTZ	Frameshift,<br>Missense	Mild	Normal ATM protein expression	Mild
**P69**	*ATM*	c.3049C>T<br>c.8083G>A	p.Gln1017*<br>p.Gly2695Cys	HTZ	Nonsense,<br>Missense	Mild	Reduction of ATM protein with kinase activity	Mild
**P71**	*ATM*	c.68G>A<br>c.6059G>A	p.Arg23Gln<br>p.Gly2020Val	HTZ	Missense,<br>Missense	Mild	Normal ATM protein expression	Mild
**Group III (Severe): + ataxic signs, + using a wheelchair**								
**P5**	NR	NR	NR	NR	NR	NR	NR	NR
**P6**	NR	NR	NR	NR	NR	NR	NR	NR
**P22**	*ATM*	c.6325T>G	p.Trp2109Gly	HMZ	Missense	Mild	Absence of ATM protein	Severe
**P30**	NR	NR	NR	NR	NR	NR	NR	NR
**P33**	*ATM*	c.9022C>T	p.Arg3008Cys	HMZ	Missense	Mild	NR	NR
**P34**	*ATM*	c.7456C>T	p. Arg2486*	HTZ	Nonsense,	Mild	NR	NR

		c.8161 G>A	p.Asp2721Asn		Missense			
**P35**	*ATM*	c.7456C>T<br>c.8161 G>A	p. Arg2486*<br>p.Asp2721Asn	HTZ	Nonsense,<br>Missense	Mild	Reduction of ATM protein	Mild
**P36**	*ATM*	IVS21+1G>A<br>IVS55+5delG	-<br>-	HTZ	Splicing,<br>Frameshift	Mild	Reduction of ATM protein	Mild
**P37**	*ATM*	IVS28-1G>C<br>IVS34+32insAlu	-<br>-	HTZ	Splicing,<br>Missense	Mild	NR	NR
**P42**	*ATM*	Dup Exon 64-65<br>c.6108T>G	-<br>p.Tyr2036*	HTZ	Frameshift,<br>Nonsense	Severe	NR	NR
**P46**	*ATM*	c.1516G>T	p.Gly506Cys	HMZ	Missense	Mild	NR	NR
**P54**	*ATM*	c.331+5G>A	-	HMZ	Splicing	Mild	Reduction of ATM kinase activity	Mild
**P55**	*ATM*	c.331+5G>A	-	HMZ	Splicing	Mild	Reduction of ATM kinase activity	Mild
**P60**	*ATM*	c.8147 T>C<br>Unidentified	p. Val2716Ala<br>Unidentified		Missense<br>-	Mild	NR	NR
**P61**	*ATM*	c.717_720delCCTC<br>c.8147 T>C	p. Leu240fs*<br>p.Val2716Ala	HTZ	Frameshift,<br>Missense	Mild	NR	NR
**P63**	*ATM*	c.496+5G>A<br>c.7875_7876delTGinsGC	p. Arg111_Glu166del55insLys<br>p.Asp2625_Ala2626delinsGluPro	HTZ	Splicing,<br>Double<br>missense	Mild	NR	NR
**P68**	*ATM*	c.6188G>A	p.Gly2063Val	HMZ	Missense	Mild	Reduction of ATM protein with kinase activity	Mild
**P70**	*ATM*	c.7024G>T<br>c(2466+1_2467-1)_(8850+1_8851-1)dup	p.Gly2342Val<br>-	HTZ	Missense,<br>Frameshift	Mild	Reduction of ATM protein with kinase activity	Mild
**P72**	*ATM*	c.496+5G>A<br>c.7875_7876delinsGC	-<br>p.Asp2625_Ala2626delinsGluPro	HTZ	Splicing<br>Double<br>missense	Mild	Reduction of ATM protein and kinase activity	Mild
**Data on using a wheelchair not available (Not included in the classification)**								
**P19**	*ATM*	c.6572ins7 (IVS47-9G>A)<br>c.5435del3	-<br>p.Ala1812Val	HTZ	Frameshift,<br>In-frame	Mild	Normal ATM protein expression	Mild

**P26**	*ATM*	c.7570G>C<br>c.3663G>A	p. Ala2524Pro<br>p.Trp1221*	HTZ	Missense, Nonsense	Mild	Reduction of ATM protein and absence of ATM kinase activity	Severe
**P27**	*ATM*	c.5189G>T<br>c.8585del87	p. Arg1730Leu<br>p.2862del29	HTZ	Missense, Frameshift	Mild	Reduction of ATM protein and the absence of ATM kinase activity	Severe
**P28**	*ATM*	c.6203T>C	p.Leu2068Ser	HMZ	Missense	Mild	Reduction of ATM protein with kinase activity	Mild
**P43**	NR	NR	NR	NR	Frameshift or Nonsense	Severe	Absence of ATM protein	Severe
**P73**	*ATM*	NR	NR	NR	NR	NR	Absence of ATM protein	Severe

ATM; ataxia-telangiectasia mutated, HMZ; homozygous, HTZ; compound heterozygous, NR: not reported.

** Based on Western blot experiment.*

*** Based on the presence or absence of protein patients were divided into two groups of severe (without residual of expression or function) and mild (with residual of expression and function)*
